# Potentially inappropriate prescribing in long-term care residents and its association with probable delirium.

**DOI:** 10.23889/ijpds.v7i3.1796

**Published:** 2022-08-25

**Authors:** Colleen Webber, Christina Milani, Christine Watt, Peter Lawlor, Genevieve Casey, Lise Bjerre, Michael Pugliese, Frank Knoefel, Franco Momoli, Kednapa Thavorn, Peter Tanuseputro

**Affiliations:** 1 Ottawa Hospital Research Institute; 2 Bruyere Research Institute; 3 Bruyere Continuing Care; 4 The Ottawa Hospital; 5 University of Ottawa; 6 ICES

## Objectives

Medications can increase the risk of delirium due to drug toxicities, polypharmacy, and drug interactions. This study examined potentially inappropriate prescribing (PIP) of medication and its association with probable delirium among long-term care residents.

## Approach

We conducted a cross-sectional study of long-term care residents in Ontario, Canada between January 1, 2016 and December 31, 2019. Routinely collected long-term care resident assessment data from the Resident Assessment Instrument – Minimum Dataset (RAI-MDS) was linked to prescription claims data to ascertain probable delirium and medication use in the two weeks preceding the index assessment. PIP was measured via the STOPP/START criteria and Beers criteria, with residents classified as having 0, 1, 2, or 3+ PIPs. Associations between PIP and probable delirium was assessed via bivariate and multivariable logistic regression models.

## Results

The study population included 171,190 long-term care residents. The mean age was 84.5 years, 66.8% were female, and 62.9% had dementia. Probable delirium was documented on 3.7% of resident assessments. Over half (51.8%) of residents had 1+ PIP and 21% had 3+ PIPs according to the STOPP/START criteria. The odds of probable delirium increased as the number of PIPs increased. Probable delirium was 1.86 times more likely (95% confidence interval 1.74-1.98) in residents with 3+ PIPs compared to those with no PIPs after confounder adjustment. Similar findings were observed when PIP was evaluated using the Beers criteria.

## Conclusion

Potentially non-beneficial interventions were common in the last 100 days of life of patients with cancer. Variations in interventions across patient demographics and cancer site may reflect differences in healthcare access, end-of-life care preferences, patients’ prognostic awareness, and disease factors.

